# The British Columbia Emergency Medicine Network: A Paradigm Shift in a Provincial System of Emergency Care

**DOI:** 10.7759/cureus.2022

**Published:** 2018-01-04

**Authors:** Riyad B Abu-Laban, Sharla Drebit, Ronald R Lindstrom, Chantel Archibald, Kim Eggers, Kendall Ho, Afshin Khazei, Adam Lund, Carolyn MacKinnon, Ray Markham, Julian Marsden, Ed Martin, Jim Christenson

**Affiliations:** 1 Department of Emergency Medicine, University of British Columbia; 2 Patient Voices Network, None; 3 Deparment of Emergency Medicine, University of British Columbia; 4 Department of Family Practice, University of British Columbia

**Keywords:** emergency medicine, network, health services, knowledge translation, quality improvement, healthcare technology, continuing professional development, clinical resources, real-time support, innovation

## Abstract

As generalists, emergency practitioners face challenges in providing state-of-the-art care owing to the broad spectrum of practice and the rapid rate of new knowledge generation. Networks have become increasingly prevalent in health care, and it was in this backdrop, and the resulting opportunity to advance evidence-informed emergency care in the Canadian province of British Columbia (BC), that a new “Emergency Medicine Network” (EM Network) was launched in 2017. The EM Network consists of four programs, each led by a physician with expertise and a track record in the domain: (1) Clinical Resources; (2) Innovation; (3) Continuing Professional Development; and (4) Real-time Support. This paper provides an overview of the EM Network, including its background, purpose, programs, anticipated evolution, and impact on the BC health care system.

## Introduction

In September 2017, a paradigm shift began in the Canadian Province of British Columbia (BC) with the launch of the BC Emergency Medicine Network (EM Network). The vision is “Exceptional emergency care. Everywhere.” and the objective of the EM Network is to improve care by sharing clinical support resources, bridging communication gaps, and strengthening relationships between clinicians across all emergency settings. The EM Network also facilitates stronger links between clinicians and researchers, policy experts, health authorities, the government, and patients. This paper provides an overview of the EM Network: its background, purpose, programs, anticipated evolution, and impact on the BC health care system.

## Technical report

Emergency medicine in BC

A recent study by the University of British Columbia (UBC) Department of Emergency Medicine found that some 1,100 physicians manage approximately two million patient visits each year in 108 emergency departments (EDs) and diagnostic and treatment centers across BC (unpublished data: Marsden J, Archibald C, Christenson J. BC emergency practitioner workforce and training survey. UBC Department of Emergency Medicine, 2016). EDs in large centers are predominately staffed by full-time emergency physicians, most certified in emergency medicine (EM) by either the Royal College of Physicians and Surgeons of Canada or the College of Family Physicians of Canada [1]. The majority of emergency practitioners in smaller BC communities consists of family physicians, who often deliver emergency care as one component of full-service primary care [2].

Regardless of their location, nature of training, or practice, all emergency practitioners are generalists, and, as such, share a generalist philosophy characterized by a commitment to the breadth of contextually appropriate care, and collaborate with the larger health care team to respond to individual patient and community needs [3].

Rationale and development of the BC Emergency Medicine Network

As generalists, emergency practitioners face challenges in providing state-of-the-art care, owing to the broad spectrum of practice and the rapid rate of new knowledge generation [4-5]. Currently, physicians and EDs struggle in isolation to define and implement management approaches for their settings and do so without formal linkage to researchers, policy experts, health authorities, government, and patient partners. Easy access to current evidence-informed best practices, in combination with the capability for real-time consultation with trusted colleagues, would help support contextually appropriate best practices, particularly for critically ill patients in smaller settings [6].

It was in this backdrop, and the resulting opportunity to advance evidence-informed emergency care in BC, that the concept of a network arose four years ago. The strategy to form the EM Network initially began within the UBC department of emergency medicine, but rapidly expanded to include numerous sponsors and partners in a shared leadership model across the BC health care system [7].

**Figure 1:**
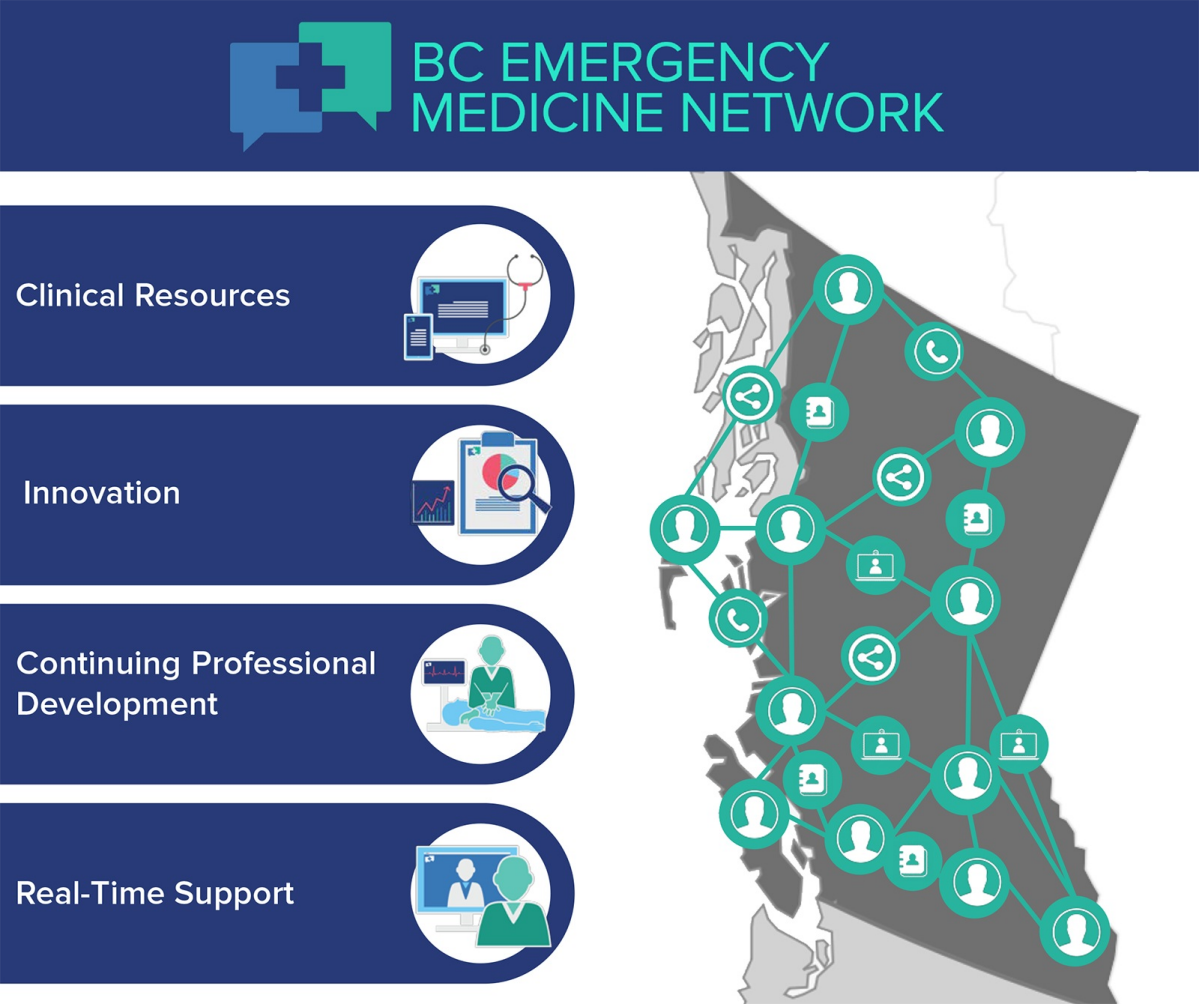
BC Emergency Medicine Network programs BC: British Columbia

An extensive repository of knowledge on all aspects of emergency care exists within the community of BC emergency practitioners. The EM Network enables multi-directional communication among physicians and the sharing of knowledge to improve emergency care and outcomes. Four programs support this mandate: Clinical Resources, Innovation, Continuing Professional Development (CPD) and Real-time Support (Figure 1). A core philosophy underscoring the EM Network is a shift away from a siloed approach to location-dependent ED care, to a collaborative approach underscored by the EM Network's mission: “Sharing, supporting and innovating to improve patient care.”

Network fundamentals and perspective

Networks have become increasingly prevalent in health care. Although networks can be conceptualized in many ways, their practical relevance largely depends on the quality and strength of their individual and organizational member relationships. Clinical networks have emerged primarily to drive innovation and improve care and patient outcomes [8]. When health professionals collaborate through a network structure, coordination, quality of care, and safety are improved [9]. BC’s health care system has seen numerous networks established, including, most recently, the BC Academic Health Science Network with which the EM Network is directly affiliated [10].

The EM Network combines key elements of public sector, inter-organizational, and clinical networks. As a result, it is substantially more than simply a communications platform or website. A growing body of scholarly and practical evidence has informed the development of the EM Network, leading to anticipated benefits that include access to resources, efficiency, quality, coordination, learning, capacity building, innovation, and responsiveness [11].

Programs of the EM Network

The EM Network consists of four programs, each led by a physician with expertise and a track record in the domain.

1. Clinical Resources

Emergency practitioners often manage clinical situations for which they have limited or no recent experience [12]. This situation is compounded by the urgency of required decisions, the solo nature of shift coverage across many settings, and the difficulty of rapidly accessing relevant current resources [4-5]. To address this, the EM Network Clinical Resources Program solicits experts to author a wide range of point-of-care clinical summaries on core emergency medical topics and has reviewed and compiled repositories of electrocardiograms (ECGs), images, procedural videos, and patient information sheets that practitioners can easily access while on shift. Physicians from across BC vet the clinical summaries to ensure their relevance and accuracy for both rural and urban practice environments. Clinical summary authors ensure their topic is regularly reviewed and updated, when necessary, and moderate discussions on the topics generated by EM Network members on the interactive platform.

2. Innovation

EM research has traditionally occurred in relative isolation, with research questions and criteria for success defined by the investigators. Both society-at-large and funders now expect research to be clinically relevant and focused on patient-oriented outcomes. The EM Network Innovation Program supports the generation and translation of new knowledge and facilitates a focus on measurable outcomes to guide policy and improvements in clinical care. The program currently includes 13 innovation initiatives on a range of topics related to clinical care, emergency systems, and prevention. Each is described by a program logic model that details the initiative’s inputs, activities, outputs, and outcomes. The website provides a unique means for EM researchers to seek collaborators or guidance on ongoing research initiatives, facilitate the knowledge translation of their research findings, and openly engage with EM Network members on interactive forums. The website also includes linkages between the Innovation and Clinical Resources Programs to break down the historical barriers and artificial separation between these activities.

3. Continuing Professional Development (CPD)

Historically, physicians maintained skills primarily through passive learning in isolation from other health care professionals and with no formal training in crisis resource management (CRM); awareness of cognitive errors and strategies to reduce them was similarly absent. As medical care has become increasingly complex, such an approach is insufficient. It is now known that medical error is a significant cause of death [13] and most often arises from communication failures. Growing evidence exists that simulated, in-situ, inter-professional team training with CRM skills results in improved patient outcomes and reduced mortality [14]. The EM Network CPD Program supports this through 1) the establishment of a unified portal for EM-related CPD registration and 2) initiatives that create and support high-quality simulation programs, strengthen regional simulation nodes across BC, and enhance the simulation curriculum for UBC emergency medicine and family medicine residents to better prepare them for entry to practice.

4. Real-time Support

An ideal emergency care system includes just-in-time peer support for physicians dealing with acute or critical cases. This support should be wide-ranging and potential examples include real-time video-conferencing to facilitate the resuscitation of a critically ill patient in a rural community, and electronic image transfer to obtain a consultation for a puzzling skin rash or to help interpret an unusual electrocardiogram. Readily available technology, such as mobile phones, text messaging, social media, and portable video-conferencing, can meet this need [15-16]. The challenge is engaging busy health professionals to establish reliable, secure, and easy approaches for real-time virtual care delivery [17]. The EM Network Real-time Support Program is currently engaged in a pilot project in Robson Valley to assess a range of virtual health scenarios. The evaluation will include patient and provider experiences, patient and family outcomes, costs, professional relationships, practice patterns, and physician remuneration. The results of this pilot will inform the development of an immediate video-linked support system across all BC EDs.

Communication strategy

The EM Network is a province-wide community of members connected through technology. The website portion of the network permits physicians to connect with online content and with one another. It acts as a portal to access and share research and best practices and encourages context-specific feedback by members on content. The website and its repositories are publicly accessible; however, only members can access discussion and feedback forums and a secure member directory that permits one-to-one or one-to-many communication. Since teasing out clinically relevant information in the modern age can be challenging [4-5], members can also choose how and when they access various information streams. For example, they can opt to receive notifications whenever a relevant activity happens on the site, receive only occasional e-mail updates, or simply interact with the site at their convenience. In this and other ways involving the filtering and highlighting of relevant information, the EM Network web platform will adapt to user-identified needs.

Patient engagement

Person-centered care has been described by the BC Ministry of Health as a way of thinking and doing things “with” patients, families, and caregivers as equal partners in health care, rather than “to” or “for” them. The EM Network is highly committed to patient engagement and, more specifically, to patients actively collaborating in governance, priority setting, and operations of the network. The EM Network will also involve patients through collaboration on the “Emergency Department Patient Experience and Patient Outcome Survey” project; an upcoming survey of over 12,000 patients who will be asked about their ED experiences.

The unique expertise and experiences of patients are highly valued and, by design, the EM Network has strived to ensure patients have an equal voice at the highest levels of decision-making. Currently, two patient partners sit on the EM Network Advisory Committee and have provided important contributions to the strategic and sustainability plan of the network and valuable input on the website and evaluation plans. These patient partners are also members of the authorship team of this paper and provide an important link to the BC “Patient Voices Network.”

Patient partners will be involved in advancing the longer term EM Network vision of expanding the number of patients and forming a patient and public council. The ultimate goal is a system where patients play a greater role in shaping emergency care in BC.

Work to date

During the past year, the EM Network established advisory and management committees that refined operational plans for the four programs and developed an evaluation framework. A three-month discovery phase commenced in late 2016 to determine the needs and perspectives of BC’s emergency practitioners. With the contracted partnership of Be The Change Group Inc*.*, we conducted 202 surveys, 25 in-depth interviews, and 14 ED site visits, many involving formal focus group discussions, at rural and urban locations across BC. The message received was consistent: “build a platform that is easy to use, allows me to connect with colleagues across BC, and gives me the ability to access information relevant to my practice.”

**Figure 2:**
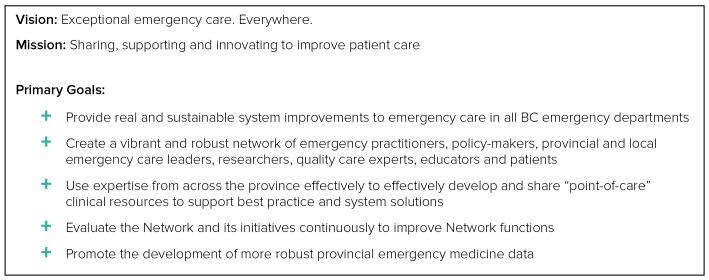
Vision, mission, and primary goals of the BC Emergency Medicine Network BC: British Columbia

**Figure 3:**
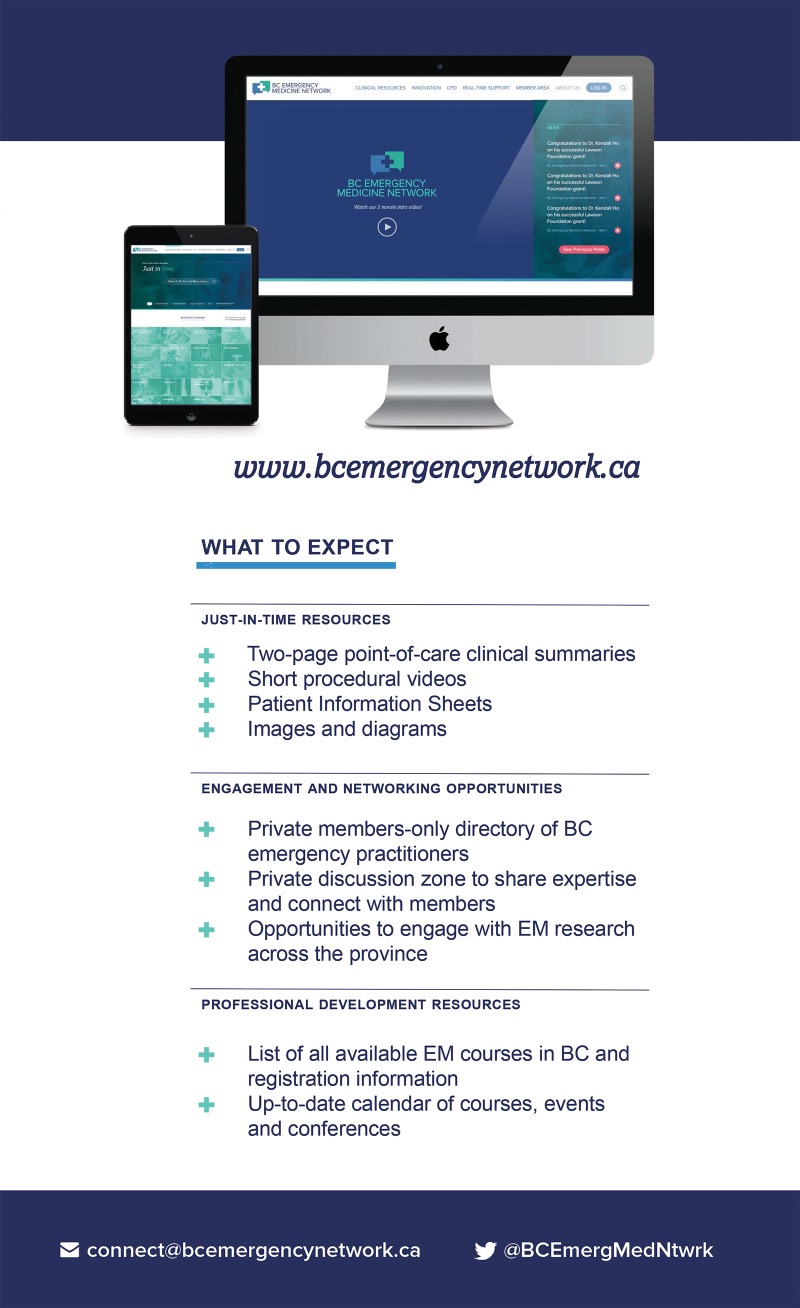
Website content of the BC Emergency Medicine Network BC: British Columbia; EM: Emergency Medicine

In early 2017, the EM Network established its brand, finalized its mission, vision, and goals (Figure 2) and developed the framework for the online platform and four EM Network programs. The beta platform was launched in July 2017. Emergency practitioners from across BC provided content and feedback throughout this process, ensuring a user-guided approach. The official launch of the EM Network occurred in September 2017 (Figure 3), and in its first 12 weeks, the EMN had already attained over 400 members. The significant membership base and content are only the beginning, however, and we look forward to frequent contributions from members to continually enhance resources and meet clinical needs.

Evaluation of the EM Network

An evaluation plan has been integrated into the design of the EM Network from its inception. Beyond assessing the network, the planned mixed methods evaluation is anticipated to help address a general lack of evidence on the effectiveness of networks, particularly as compared to control groups [18]. The quantitative component of the evaluation will utilize social network analysis, specifically the “Program to Analyze, Record, and Track Networks to Enhance Relationships” (PARTNER) tool [19] to generate metrics regarding progress on the EM Network's structure, function, and outcomes. Relationships over time will be mapped and analyzed to evaluate progress and determine if changes are indicated. The qualitative component of the evaluation will include semi-structured interviews and focus groups with EM Network members province-wide. This will generate findings related to four key questions:

(1) What is the Network trying to accomplish?

(2) Is the Network organized appropriately?

(3) Are Network members working well together?

(4) Are Network members supported in their role?

Additional evaluations will include patient and systems outcomes, patient engagement, website analytics, and specific innovation initiatives and research projects supported by the EM Network.

Generalizability and cost of the EM Network

We believe our experience in establishing the EM Network is generalizable to other Canadian provinces or US states. Development of the EM Network required significant financial and human investment; three years were spent developing the concept and engaging with partners and stakeholders to grow enthusiasm and secure funding. Travel around BC was essential to build relationships, assess needs, obtain input, and validate the concepts and style with future members. Many leaders adjusted their academic activities to put an emphasis on building the EM Network. Innovation leads had funding from various sources prior to the EM Network. Others without protected time were provided small honoraria to collate content for the launch. We believe our securing of an EM Network manager and administrative support was essential to our success. We engaged a professional company to build a state-of-the-art and functional website to hold the content and facilitate member engagement and communication. Following the website build, expenses have continued. To build and maintain the EM Network, including management, clinical support, and evaluation (excluding academic researcher salaries and real-time functions) cost approximately $725,000 in the first year and is projected to cost approximately $575,000 in each subsequent year.

## Discussion

It is anticipated that the BC Emergency Medicine Network will improve clinical care and patient outcomes in BC by efficiently providing relevant point-of-care clinical resources, supporting new clinical and research collaborations, enhancing continuing professional development, and building a system of real-time support. The Network also has the potential to help address broader system issues in emergency care, such as the access block of EDs by admitted patients and improving the recruitment and retention of emergency practitioners in rural settings [20]. Although initially launched with a membership focus on physicians, the EM Network may evolve to meet the needs of other emergency clinicians, such as emergency nurses, nurse practitioners, and paramedics, and may evolve to partner beyond the borders of BC.

## Conclusions

The EM Network is a new and systematic approach to improving emergency care and will require full engagement by emergency practitioners in BC. Sharing knowledge, discussing needs, and solving common problems are essential to the successful growth and development of the EM Network and, thus, essential to improvement in emergency care. Ultimately, the true measure of the success of the EM Network will be its impact on patients in EDs across British Columbia.
